# Causal relationship between complement *C1QB* and colorectal cancer: a drug target Mendelian randomization study

**DOI:** 10.3389/fgene.2024.1403509

**Published:** 2024-07-23

**Authors:** Mingwen Jiao, Yuying Cui, Xiaodong Qiu, Xuezhen Liang, Junhan Li, Congcong Guo, Hu Tian

**Affiliations:** ^1^ Department of General Surgery, Shandong Provincial Qianfoshan Hospital, Shandong University, Jinan, Shandong, China; ^2^ The First Clinical Medical College, Shandong University of Traditional Chinese Medicine, Jinan, Shandong, China; ^3^ Central Hospital Affiliated to Shandong First Medical University, Jinan, Shandong, China; ^4^ Shandong First Medical University and Shandong Academy of Medical Sciences, Jinan, Shandong, China; ^5^ Shandong University of Traditional Chinese Medicine, Jinan, Shandong, China; ^6^ Department of General Surgery, The First Affiliated Hospital of Shandong First Medical University and Shandong Provincial Qianfoshan Hospital, Key Laboratory of Metabolism and Gastrointestinal Tumor, Key Laboratory of Laparoscopic Technology, Shandong Medicine and Health Key Laboratory of General Surgery, Jinan, China

**Keywords:** *C1QB*, colorectal cancer, drug target Mendelian randomization, molecular docking, phenotype scanning

## Abstract

**Background:**

Colorectal cancer is influenced by several factors such as unhealthy habits and genetic factors. *C1QB* has been linked to a number of malignancies. However, uncertainty surrounds the connection between *C1QB* and CRC. Therefore, this study aimed to explore a bidirectional causal relationship of *C1QB* as a drug target in CRC through Mendelian randomization (MR) analysis.

**Methods:**

The GWASs for *C1QB* and CRC were obtained from the Integrative Epidemiology Unit Open GWAS database. There were five strategies to investigate MR. Sensitivity analysis was carried out via tests for heterogeneity, horizontal pleiotropy and leave-one-out effects to evaluate the dependability of the MR analysis results. Furthermore, colocalization analysis of *C1QB* and CRC, protein-protein interaction network and drug prediction according to exposure factors as well as phenotype scanning were performed.

**Results:**

The results of forward MR analysis demonstrated that *C1QB* was a risk factor for CRC (OR = 1.104, *p* = 0.033). However, we did not find a causal relationship between CRC and *C1QB* (reverse MR). Rs294180 and rs291985 corresponded to the same linkage interval and had the potential to influence *C1QB* and CRC, respectively. The PPI results demonstrated that *C1QB* interacted with 10 genes (*C1QA*, *C1QC*, *C1R*, *C1S*, *C2*, *C4A*, *C4B*, *CALR*, *SERPING1*, and *VSIG4*). Additionally, 21 medications were predicted to match *C1QB*. Molecular docking data, including for benzo(a)pyrene, 1-naphthylisothiocyanate, calcitriol and medroxyprogesterone acetate, revealed excellent binding for drugs and proteins. Moreover, we identified 29 diseases that were associated with *C1QB* and related medicines via disease prediction and intersection methods. As a therapeutic target for CRC, phenotypic scanning revealed that *C1QB* does not significantly affect weight loss, liver cirrhosis, or nonalcoholic fatty liver disease, but might have protective impacts on ovarian cancer and melanoma.

**Conclusion:**

The results highlight a causal relationship between *C1QB* and CRC and imply an oncogenic role for *C1QB* in CRC, as potential drug targets. Drugs designed to target *C1QB* have a greater chance of success in clinical trials and are expected to help prioritize CRC drug development and reduce drug development costs. That provided a theoretical foundation and reference for research on CRC and *C1QB* in MR.

## 1 Introduction

According to the GLOBOCAN report, colorectal cancer (CRC) ranks third in incidence among malignant tumors and is the second leading cause of cancer death worldwide, affecting both genders (Sung et al., 2021). The variables associated with CRC risk include obesity, unhealthy dietary habits, smoking, aging, chronic inflammation and genetic predisposition (Hossain et al., 2022). In 2020, more than 1.9 million new colorectal cancer cases were diagnosed each year, with approximately 930,000 deaths globally. The burden of CRC is projected to increase to 3.2 million new cases and 1.6 million deaths by the year 2040 (Morgan et al., 2023). Despite existing treatment for CRC patients have achieved great advances, such as in surgery, chemotherapy, radiotherapy, molecularly targeted therapy and immunotherapy, barriers remain: high postoperative recurrence, chemotherapy drug resistance, and severe adverse effects limit therapeutic efficacy and result in poor prognosis. Therefore, further searching for potential biomarkers and treatment methods for CRC is crucial for enhancing the detection and longevity of individuals with CRC.

Complement is a class of peptides with enzyme activity and self-regulation. And the complement system represents an important component of the inflammatory response, which regulates both innate and adaptive immune responses. C1q is the first subcomponent of the complement classical pathway, which can activate the complement cascade when associated with C1r and C1s (Lu and Kishore, 2017). Human C1q is a hexameric molecule, assembled from 18 polypeptide chains of three different types, A (28 kDa), B (25 kDa), and C (24 kDa). Each of the three polypeptide chains consists of a C-terminal globular domain (gC1q), linked to a N-terminal collagen-like domain (cC1q) (Nayak et al., 2012). The role of the cC1q mainly mediates the immune effect by interacting with C1r and C1s proteases to activate the complement. And the gC1q domain is responsible for target recognition. It includes binding to the Fc region of immunoglobulin, recognition of surface proteins of bacteria and viruses (Kishore et al., 2004). In addition to binding IgG and IgM containing immune complexes and activating the complement classical pathway, there is emerging evidence to suggest that C1q plays crucial role in a wide range of physiological and pathological processes, such as placental development (Agostinis et al., 2017a), wound healing (Bossi et al., 2014), autoimmunity (Son et al., 2015) and cancer (Ghebrehiwet et al., 2024). C1q is locally synthesized by macrophages and dendritic cells, and has recently entered the limelight due to its immunoregulatory functions in the tumor microenvironment (TME). The notion of C1q involvement in the pathogenesis of cancer is still evolving. C1q appears to exert a dual role in cancer: tumor promoting as well as tumor-protective, depending on types of human tumors. Bioinformatics analysis in various carcinomas reported that high levels of C1q have a favorable prognostic index in basal-like breast cancer (Mangogna et al., 2019a), HER-2 positive breast cancer (Mangogna et al., 2019a), and skin cutaneous melanoma (Liang et al., 2022; Yang et al., 2022). However, C1q paly a pro-tumorigenic role in lung adenocarcinoma (Mangogna et al., 2019a), clear cell renal cell carcinoma (CCRCC) (Mangogna et al., 2019a) and Glioma (Mangogna et al., 2019b). *C1QA, C1QB* and *C1QC* genes encode the three C1q chains, respectively. Although the three genes are interdependent and often transcribed synchronously (Chen et al., 2011). Their expression levels seem to be not completely consistent across different pathological types of cancer. For example, the expression of the three C1q chains was higher in CCRCC as compared to normal kidney. However, in the case of papillary renal cell carcinoma (PRCC), this trend was evident only for *C1QA* and *C1QB* mRNA expression (Mangogna et al., 2019a). Junjie Jiang et al. reported that higher mRAN expression of *C1QB*, instead of *C1QA* and *C1QC* was in gastric cancer tissues than that in adjacent normal tissues (Jiang et al., 2020). The iTRAQ-based quantitative proteomic analysis reveals that the protein expression levels of C1QB and C1QC were upregulated in serum sample of patients with CCRCC and were significantly associated with the grade and stage of CCRCC (Zhang et al., 2016). Recently, Huiming Deng et al. showed that the protein expression of C1QC in CRC tissues was higher than that in non-cancer tissue by immunohistochemical staining. And a high protein expression of C1QC in CRC patients led to a poor prognosis (Deng et al., 2022). However, the associations between *C1QB* and the prognosis of CRC remain unclear due to insufficient sample size.

**FIGURE 1:**
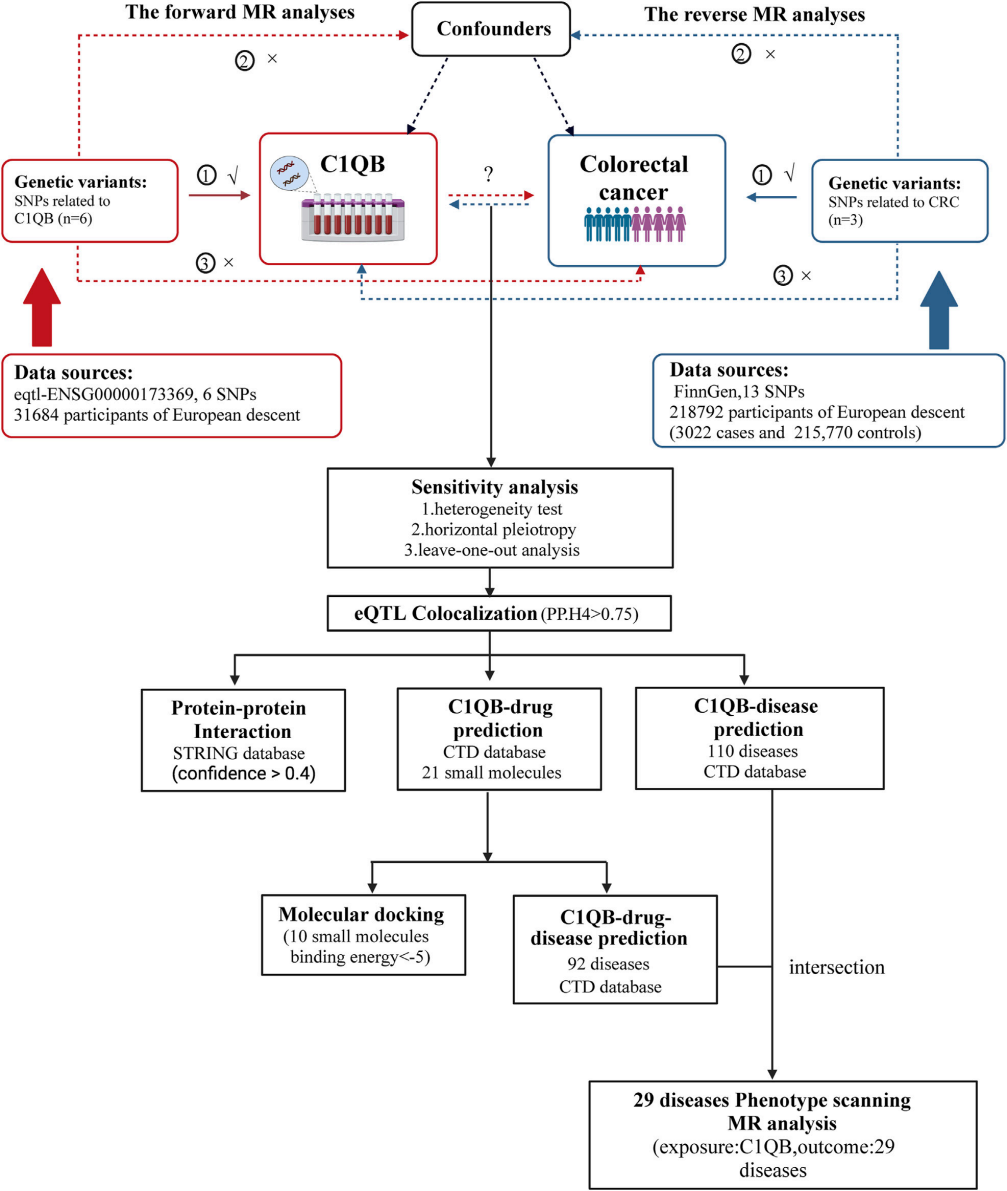
Overview of the study design. MR analyses depend on three core assumptions: ①Relevance (G is associated with the X).②Independence (G is not related to any confounding factors of the exposure-outcome association).③Exclusion restriction (G does not affect Y except through its potential effect on the X. The red represented the forward MR analyses, with *C1QB* as exposure and CRC as outcome. The blue represented the reverse MR analyses, with CRC as exposure and *C1QB* as outcome. Abbreviations: CRC, Colorectal cancer; MR, Mendelian randomization; SNPs, single-nucleotide polymorphisms.

The statistical technique of Mendelian randomization (MR) has emerged as a robust method for evaluating causal connections through the use of genetic variables as instrumental factors in recent years (Holmes et al., 2017). Owing to the abundance of summary data from genome-wide association studies (GWAS), MR can be used to effectively and affordably determine the causal link between exposure and disease outcome (Liu et al., 2021). Drug-targeting MRs are new research designs that can be developed for drug repurposing by employing genetic tools near or within target genes to mimic the potential effects and dangers of drug targets. In the present study, we utilized a bidirectional two-sample MR design and used data from GWASs, to examine the causal relationship between *C1QB* and CRC. Subsequently, co-localization analysis of the *C1QB* gene and colorectal cancer tissue was performed to identify the expression quantitative trait locus (eQTL) associated with colorectal cancer. Finally, a protein-protein interaction (PPI) network was constructed, and drug prediction according to exposure factors and phenotype scanning were performed to provide new ideas for drug development in colorectal cancer. The study design is shown in Figure 1 **.**

## 2 Methods

### 2.1 Data preprocessing

The GWASs for colorectal cancer (CRC) (finn-b-C3_COLORECTAL) and C1QB (eqtl-a-ENSG00000173369) were obtained from the Integrative Epidemiology Unit (IEU) Open GWAS database (https://gwas.mrcieu.ac.uk/). The CRC dataset included 16,380,466 single nucleotide polymorphisms (SNPs). There were 18,308 SNPs and 31,684 samples for *C1QB*. Utilizing the “Two Sample MR” R program (version 4.3.1), SNPs (instrumental variables (IVs)) were subsequently chosen (forward Mendelian randomization (MR): *p* < 5*10^8; reverse MR: *p* < 5*10^6) (Huang et al., 2023). Six independent SNPs closely associated with *C1QB* and 3 independent SNPs closely associated with CRC were subsequently filtered out (Supplementary Table S1). In forward MR, IVs of the exposure factor (*C1QB*, clump = TRUE, instrumental variable for linkage disequilibrium (LD) removed; r^2^ = 0.001; kb = 10,000) and outcome (CRC, proxy = TRUE; rsq = 0.8) were screened separately. The same procedure was applied for screening in reverse MR images. The dataset GSE87211, which is associated with colorectal cancer, was obtained from the Gene Expression Omnibus data database, and box-and-line plots were drawn to determine the differential expression of *C1QB*.

### 2.2 MR and sensitivity analysis

The three underlying hypotheses that served as the foundation for the MR investigation were as follows: 1) that there is a strong and remarkable relationship between IVs and exposure factors; 2) that IVs are not affected by confounders; and 3) that the impact of IVs on outcomes is solely determined by exposure factors, not other channels. To investigate the connection between *C1QB* and CRC, we used bidirectional MR analysis. There were five strategies to investigate MR: MR- Egger, inverse variance weighted (IVW), weighted median, simple mode and weighted mode. *p* < 0.05 was considered to indicate statistical significance in the MR analyses. Then, odds ratios (ORs) were computed, with ORs larger than 1 denoting a hazardous factor and ORs less than 1 denoting a protective factor. Scatter plots, forest plots, and funnel plots were created to display the results. Sensitivity analysis was carried out via tests for heterogeneity, horizontal pleiotropy and leave-one-out (LOO) effects to evaluate the dependability of the MR analysis results.

### 2.3 Bayesian colocalization analysis

Bayesian colocalization analyses were used to assess the probability that two traits share the same causal variant using the “coloc” package (https://github.com/chr1swallace/coloc) with default arguments. To clarify how significant signaling loci affect outcomes, “coloc” was used to analyze the colocalization of CRC cells with *C1QB*. The joint analysis involves four hypothetical scenarios: The first scenario H0 suggests that phenotype 1 (GWAS) and phenotype 2 (e.g., eQTL) are not significantly associated with all SNP loci in a genomic region. The second scenario H1/H2 suggests that either phenotype 1 (GWAS) or phenotype 2 (e.g., eQTL) is significantly associated with SNP loci in a genomic region. The third scenario H3 suggests that both phenotype 1 (GWAS) and phenotype 2 (e.g., eQTL) are significantly associated with SNP loci in a genomic region, driven by different causal variant loci. The fourth scenario H4 suggests that both phenotype 1 (GWAS) and phenotype 2 (e.g., eQTL) are significantly associated with SNP loci in a genomic region, driven by the same causal variant locus. Based on these four scenarios, we aim to establish a higher probability for the fourth scenario H4 in statistical terms, enabling an explanation of how significant signal loci affect phenotypes. H0, H1/H2, H3, and H4 indicate that phenotypes 1 (GWAS) and two (in the case of eQTLs) are not substantially correlated with each other, are significantly correlated with each other but driven by distinct causal variant loci, are significantly correlated with each other and are driven by the same causal variant locus, respectively. The threshold of significance for colocalization was set at PP.H4 >0.75. The colocalization results were visualized via the “gwasglue” and “gassocplot” R programs (version 4.3.1).

### 2.4 Protein-protein interaction (PPI) network construction and drug prediction

Moreover, the protein-protein interaction (PPI) network of *C1QB* was constructed using a tool to search for recurring instances of neighboring genes (STRING, https://string-db.org) (confidence > 0.4). The Comparative Toxicogenomics Database (CTD, http://ctdbase.org/) was utilized to predict drugs that bind to *C1QB*. Cytoscape was used to display the PPI and drug prediction findings.

### 2.5 Molecular docking

To further understand the effect of drug small molecules on *C1QB* chain and the druggability of target genes, this study further performed molecular docking at the atomic level. The 3D structures of the drugs were obtained from the PubChem Compound Database (https://pubchem.ncbi.nlm.nih.gov/), after which the energy needed to minimize further docking was minimized. The structure of *C1QB* chain (PDB ID:2wnv) was downloaded from the PDB (Protein Data Bank, http://www.rcsb.org/). The target includes ligand and water removal, hydrogen addition, and amino acid optimization and patching and were saved in pdbqt format. We subsequently used Autodock Vina 1.2.0 (http://autodock.scripps.edu/), a computerized protein–ligand docking software package, to examine and confirm the strength of the bond between the compound and target. The binding models were visualized with PyMol 2.3.0 software and Discovery Studio 3.5 software.

### 2.6 Disease prediction and phenotype scanning

The thresholds were set at an inference score >50 to predict diseases connected to *C1QB* and *C1QB*-drug via the CTD, respectively. The intersecting diseases were identified via a Venn diagram to pinpoint the intersection of *C1QB*-related and *C1QB* drug-related diseases. Finally, the intersecting diseases were identified in the GWAS database, and MR analysis was carried out with *C1QB* as the exposure factor and intersecting diseases as the outcome. In the present MR analysis, the inverse variance weighted (IVW) method was used as the primary analysis to combine the variant-specific Wald estimators by taking the inverse of their approximate variances as the corresponding weights.

## 3 Results

### 3.1 The causal effect of *C1QB* on CRC

**FIGURE 2:**
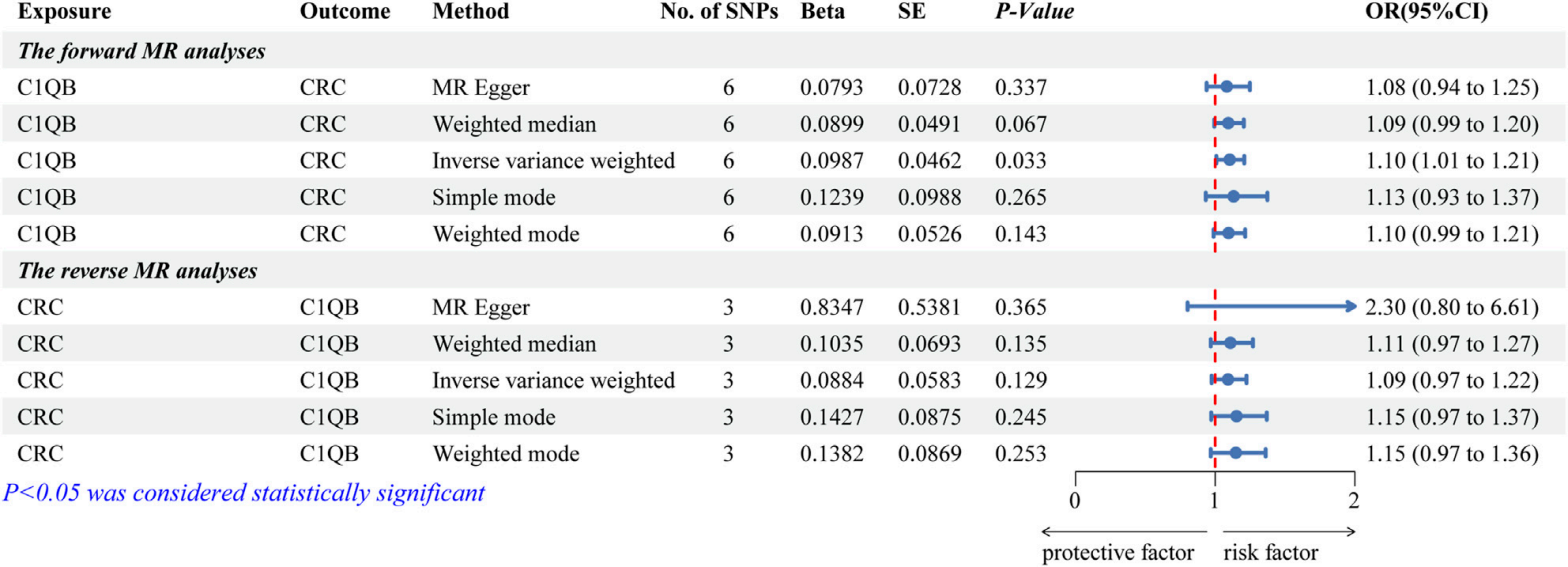
Forest plot of fiveMR estimators of the association between *C1QB* and CRC. Abbreviations: CRC, Colorectal cancer; MR, Mendelian randomization; OR, odds ratio; CI, confidence interval; SNPs, single-nucleotide polymorphisms.

After screening, we selected six independent SNPs as IVs. The forward MR analyses are listed in Supplementary Table S2. The IVW findings revealed that *C1QB* (*p* = 0.033) was causally associated with CRC. Tracking of the ORs (OR = 1.104) revealed that *C1QB* was a risk factor for CRC (Figure 2). The other models did not reveal any significant associations. The scatter plot illustrated that the *C1QB* line’s slope was positive, indicating that an increase in *C1QB* led to an increased risk of CRC (Supplementary Figure S1A). According to the forest plot, when the MR effect size exceeded 0, *C1QB* was a risk factor for CRC (Supplementary Figure S1B). Additionally, the funnel plot showed that the forward-looking MR images adhered to Mendel’s second law of random grouping (Supplementary Figure S1C).

### 3.2 The causal effect of CRC on *C1QB*

After the screening of IVs, a total of 3 IVs (SNPs) were obtained. The reverse MR analyses are listed in Supplementary Table S3. Figure 2 illustrates that none of the five algorithms were statistically significant. The scatter, forest, and funnel diagrams of the reverse MR images also confirmed the IVW results (Supplementary Figures S1D–F).

### 3.3 Sensitivity test

Several tests were conducted to assess the precision of the analysis. There was no indication of heterogeneity in the sample, according to the Q-value of the heterogeneity test (for both forward and reverse MR images, the heterogeneity was greater than 0.05) (Supplementary Table S4). Similarly, there was no impact of horizontal pleiotropy between *C1QB* and CRC, according to the results of the test (forward MR: *p* = 0.747; reverse MR: *p* = 0.396) (Supplementary Table S4). LOO analysis supported IVW and demonstrated the accuracy of the MR analysis (Supplementary Figure S2).

### 3.4 Co-localization analysis

**FIGURE 3:**
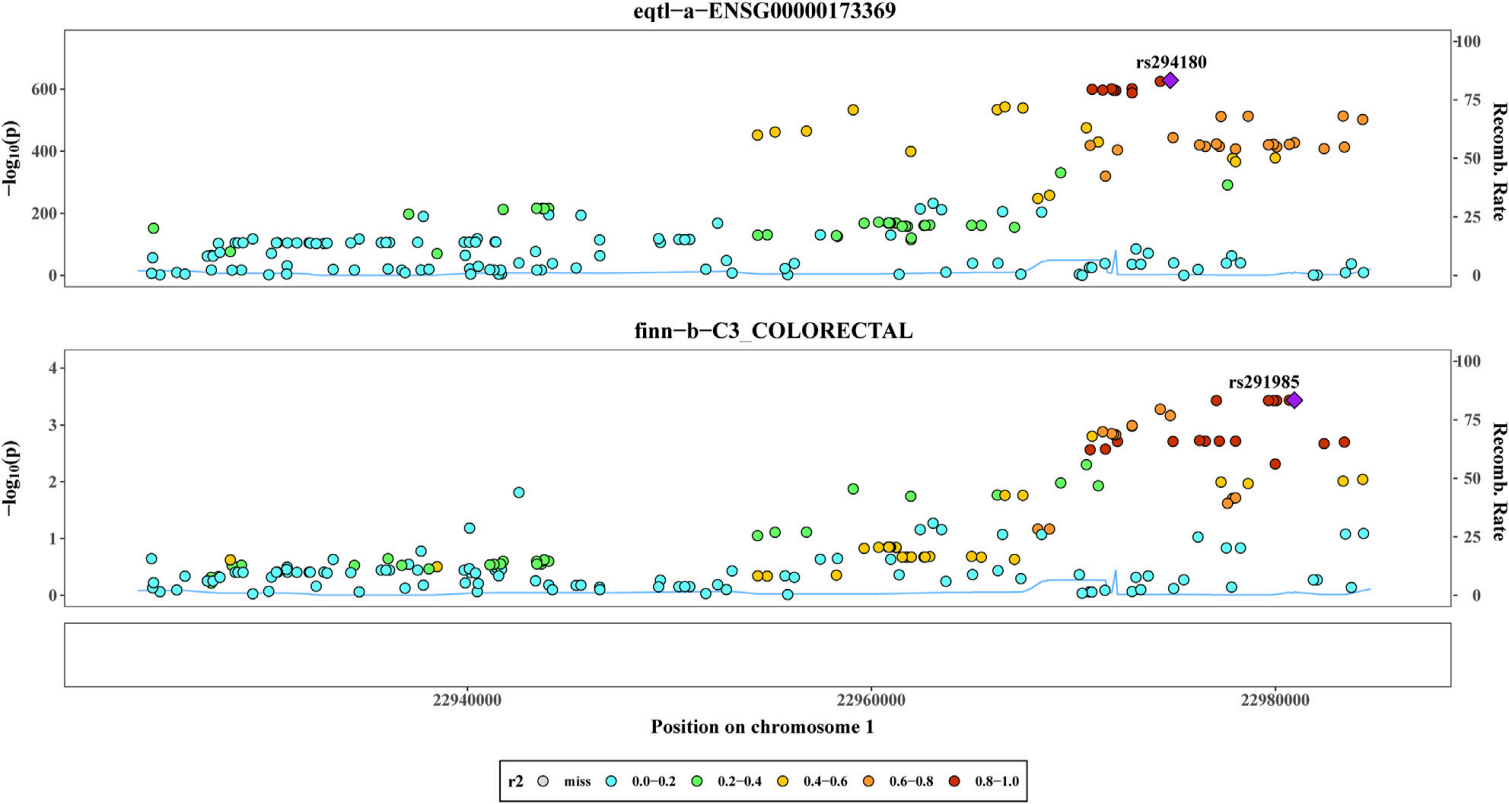
Co-localization analysis. The Results of co-localisation analysis based on eqtl−a−ENSG00000173369 and finn−b−C3_COLORECTAL with different coloured circles representing Recomb Rate.

By utilizing co-localization analysis, researchers can determine whether a particular SNP is responsible for both exposure and outcome, as long as the SNP is strongly linked to both variables. Studies have shown that proteins subjected to MR and colocalization testing may serve as potential drug targets. According to the findings of the colocalization study, there was a 78.08% probability that two characteristics (*C1QB* and CRC) were impacted by interlocking SNP mutations. Rs294180 and rs291985 corresponded to the same linkage interval and had the potential to influence *C1QB* and CRC, respectively (Figure 3). In addition, we did gene expression level analysis of *C1QB* in CRC and normal groups based on the dataset GSE87211, and found that *CIQB* expression was higher in the normal group (Supplementary Figure S3).

### 3.5 PPI network construction and drug forecasting

**FIGURE 4:**
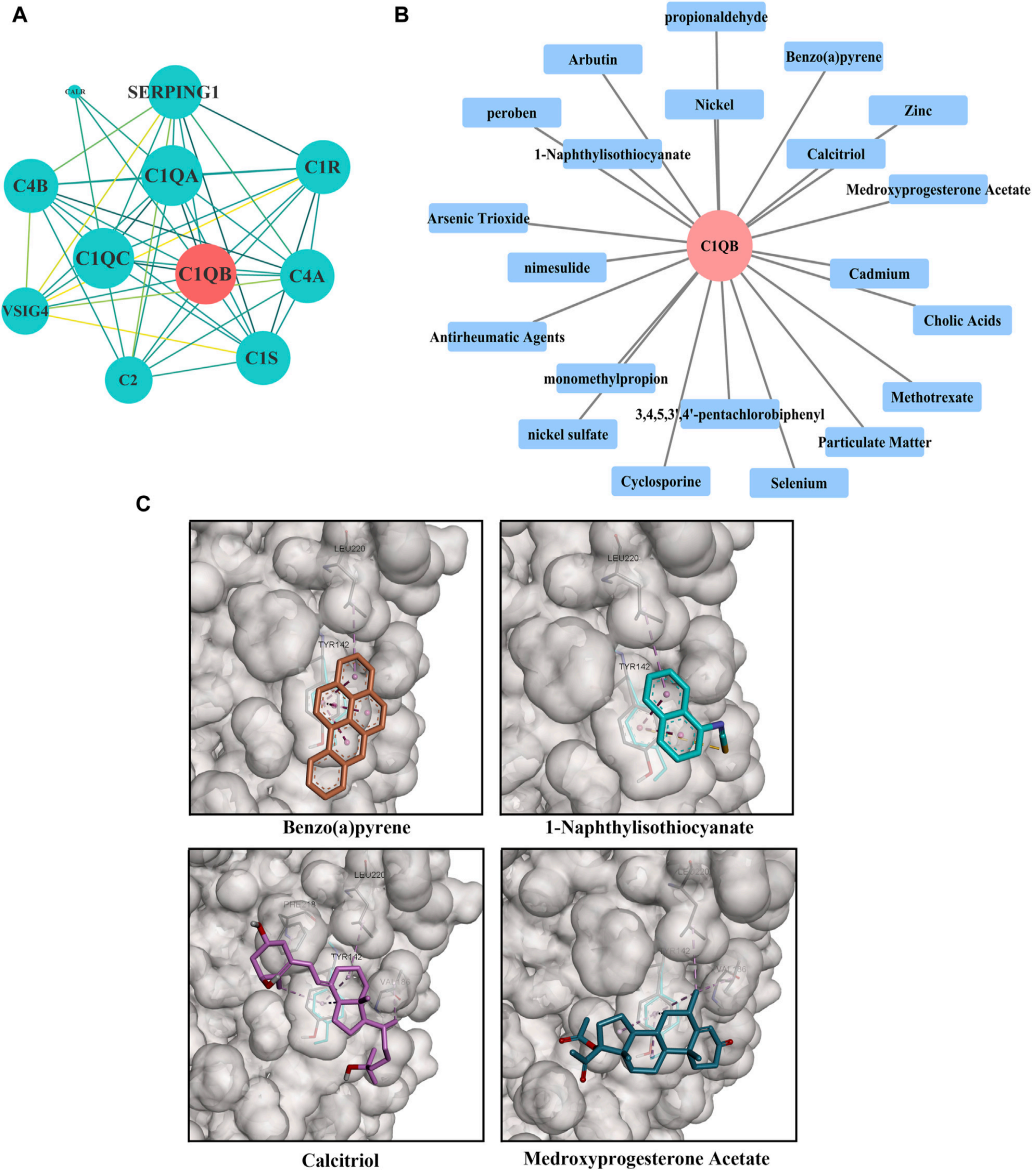
**(A)** PPI network built with STRING. The red circles represent *C1QB*, and the green rectangles represent the associated genes **(B)** Drug prediction results of *C1QB*. The red circle represents genes, and the blue rectangle represents drugs **(C)** Docking results of small molecules. *C1QB* docking Benzo(a)pyrene, *C1QB* docking 1-Naphthyl isothiocyanate, *C1QB* docking calcitriol, *C1QB* docking medroxyprogesterone acetate. The four drugs all mainly interacted with the residues TYR:142 and LEU:220.

*C1QB* was loaded into the STRING (https://cn.string-db.org/) database for network creation, and the resulting files were imported into Cytoscape for visualization. As shown in Figure 4A, the PPI analysis demonstrated that *C1QB* interacted with 10 proteins (*C1QA*, *C1QC*, *C1R*, *C1S*, *C2*, *C4A*, *C4B*, *CALR*, *SERPING1*, and *VSIG4*), whereas *C1QA* and *C1QC* interacted most strongly with *C1QB*. Drug prediction results demonstrated that twenty-one medications were associated with *C1QB* (Figure 4B). The binding affinity of the drug candidates for *C1QB* chain was assessed through molecular docking to gain a better understanding of the potential of the drug target for being targeted by drugs. The binding sites and interactions of the ten drug candidates with *C1QB* chain, which are encoded by their respective genes, were obtained using AutoDock Vina v.1.2.2. The binding energy for each interaction was also generated, achieving valid docking results using the prescribed drugs (Table 1). The greater the absolute value of the docking affinity was, the stronger the binding ability between the compound and the active site of the target was. According to the docking data, most of the binding complexes exhibited high binding affinities, with an average of −6.85 kcal/mol. The modes of the top four binding complexes are displayed in Figure 4C, and included *C1QB*-Benzo(a)pyrene docking (−9.5 kcal/mol), *C1QB*-1-naphthyl isothiocyanate docking (−7.3 kcal/mol), *C1QB*-calcitriol docking (−7.2 kcal/mol), and *C1QB*-medroxyprogesterone acetate (−7.2 kcal/mol). Benzo(a)pyrene exhibited the lowest binding energy, indicating extremely stable binding. Based on the molecular docking simulation results, four drugs that fit precisely into the same binding pocket of the target protein were observed. The four drugs all mainly interacted with the residues TYR:142 and LEU:220.

**TABLE 1:** Docking results of available *C1QB* with small molecules.

Chemical name	Formula	PubChem CID	Binding energy	Molecular weight	H-bond acceptor	H-bond donor	AlogP	Smile
Benzo(a)pyrene	C_20_H_12_	2,336	−9.5	252.32	0	0	5.74	C1 = CC = C2C3 = C4C(=CC2 = C1)C=CC5 = C4C(=CC = C5)C=C3
1-Naphthyl isothiocyanate	C_11_H_7_NS	11,080	−7.3	185.25	2	0	3.57	C1 = CC = C2C(=C1)C=CC = C2N = C=S
Calcitriol	C_27_H_44_O_3_	5,280,453	−7.2	416.65	3	3	5.7	CC(CCCC(C)(C)O)C1CCC2C1(CCCC2 = CC = C3CC(CC(C3 = C)O)O)C
Medroxyprogesterone Acetate	C_24_H_34_O_4_	6,279	−7.2	386.53	4	0	4.66	CC1CC2C(CCC3(C2CCC3(C (=O)C)OC(=O)C)C)C4(C1 = CC(=O)CC4)C
3,4,5,3′,4′-Pentachlorobiphenyl	C_12_H_5_Cl_5_	63,090	−6.7	326.44	0	0	6.62	C1 = CC(=C(C=C1C2 = CC(=C(C(=C2)Cl)Cl)Cl)Cl)Cl
Nimesulide	C_13_H_12_N_2_O_5_S	4495	−6.6	308.31	5	1	2.76	CS(=O)(=O)NC1 = C(C=C(C=C1)[N+](=O)[O-])OC2 = CC = CC = C2
Peroben	C_26_H_34_N_2_O_3_	192,144	−6.6	422.57	4	1	4.36	CCC1(C (=O)C=CNC1 = O)CC.CN(C)CCOC(C1 = CC = CC = C1)C2 = CC = CC = C2
Cholic Acids	C_24_H_40_O_5_	221,493	−6	408.58	4	4	3.45	CC(CCC(=O)O)C1CCC2C1(C(CC3C2C(CC4C3(CCC(C4)O)C)O)O)C
Monomethylpropion	C_10_H_13_NO	1,576	−6	163.22	2	1	1.48	CC(C (=O)C1 = CC = CC = C1)NC
Arbutin	C_12_H_16_O_7_	440,936	−5.4	272.25	7	5	−1.43	C1 = CC(=CC = C1O)OC2C(C(C(C(O2)CO)O)O)O

The lower the Binding Energy, the better the binding effect and the higher the affinity.

### 3.6 Role of *C1QB* in other diseases

**FIGURE 5:**
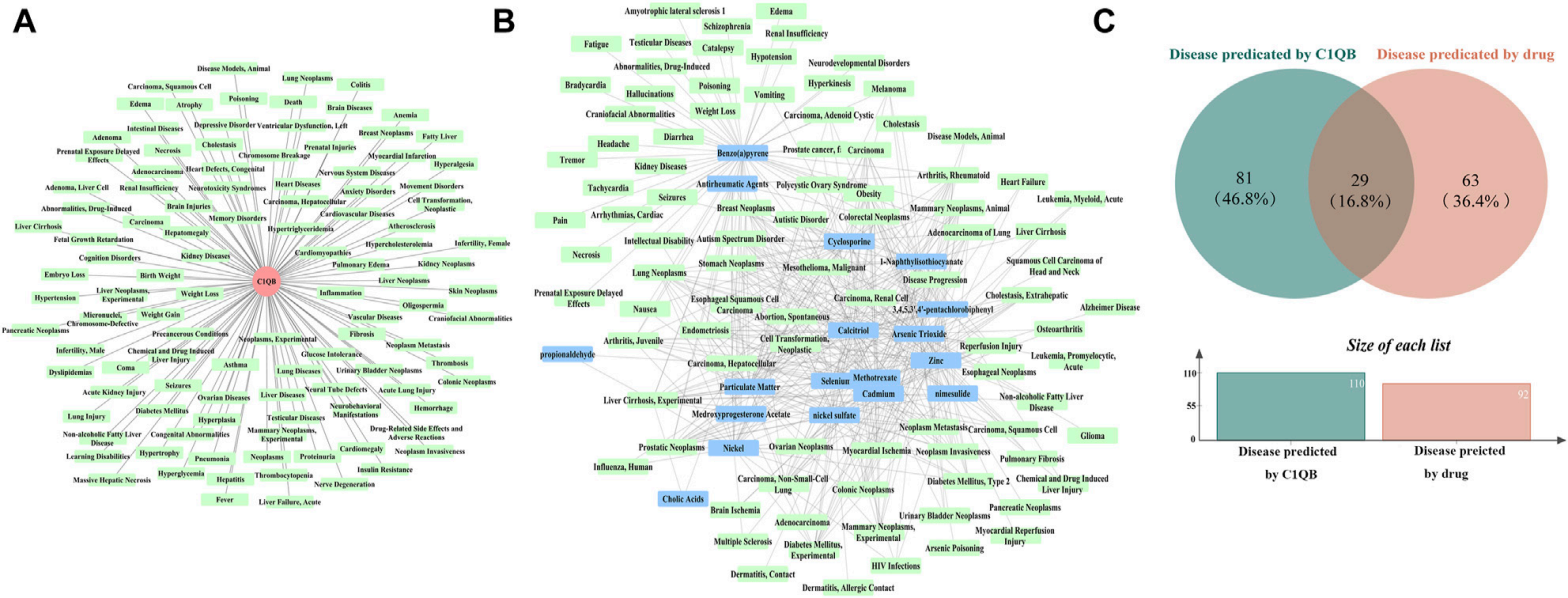
**(A)** Disease prediction results for *C1QB*. Red circles represent *C1QB*, green rectangles represent diseases **(B)** Disease prediction results of drugs. Green rectangles represent diseases, blue rectangles represent drugs **(C)** Venn diagram intersection of *C1QB* predicted diseases and drug predicted disease.

**FIGURE 6:**
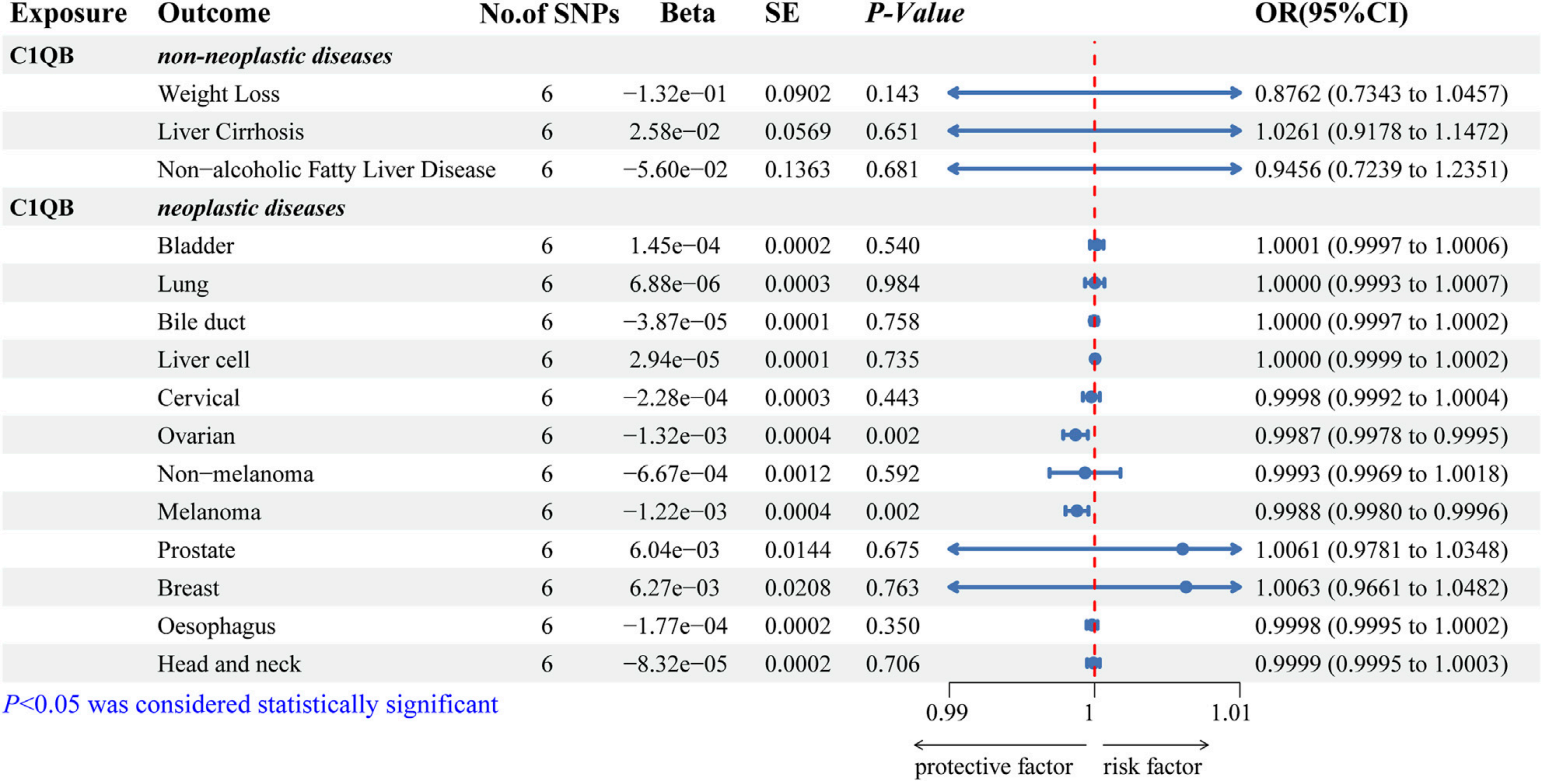
Associations of genetically predicted *C1QB* with predicted diseases. Abbreviations: CRC, Colorectal cancer; MR, Mendelian randomization; OR, odds ratio; CI, confidence interval; SNPs, single-nucleotide polymorphisms.

We predicted 110 and 92 *C1QB*-related and *C1QB* drug-related diseases, respectively, with 29 diseases satisfying both the *C1QB* and *C1QB*-drug associations (Figures 5A–C). Using the GWAS database, and searching for GWAS id data related to 29 diseases, not all the diseases could be searched for relevant data. Based on the data retrieved, only weight loss, liver cirrhosis and nonalcoholic fatty liver disease were found among the nonneoplastic disease patients. We subsequently conducted Mendelian randomization analysis of *C1QB* for the three diseases, As shown in Figure 6, MR analyses revealed no causal role of *C1QB* in weight loss, liver cirrhosis or nonalcoholic fatty liver disease risk. MR analysis was subsequently performed for *C1QB* and 12 other cancers. Using the random model IVW, we found that *C1QB* seems to be negatively associated with ovarian cancer (OR = 0.9987, 95% CI = 0.9992–1.0003, *p* < 0.0023) and melanoma cancer (OR = 0.9988, 95% CI = 0.9980–0.9996, *p* < 0.0024) risk. Therefore, we conclude that as a therapeutic target for CRC, phenotypic scanning revealed that *C1QB* does not significantly affect weight loss, liver cirrhosis, or nonalcoholic fatty liver disease, but might have protective impacts on ovarian cancer and melanoma.

## 4 Discussion

This study indicated that *C1QB* was a risk factor for CRC based on several MR methods (IVW, MR-Egger, weighted median, simple mode, weighted mode, horizontal multiplicity test and Cochran’s Q heterogeneity test), which are capable of eliminating any influencing variables during measurement. Additionally, colocalization served as significant supporting evidence. The above results guarantee and explain the reliability of the MR analysis results. By pinpointing small-molecule drugs with exceptional binding strength and beneficial interaction characteristics, we can prioritize four drugs (benzo(a)pyrene, 1-naphthyl isothiocyanate, calcitriol and medroxyprogesterone acetate) as specific target drugs for *C1QB* chain for further experimental validation. Additionally, a phenome-wide association analysis was conducted to demonstrate the potential pleiotropy of the target gene and the possible side effects of the drugs.

*C1QB*, together with *C1QA* and *C1QC*, controls the protein translation of C1q. *C1QA*, *C1QB*, and *C1QC* are three genes with strong interactions (Chen et al., 2011). Our PPI network also confirmed this point. Currently, many bioinformatics analyses have supported the impact of the three genes on tumor development and play protective or harmful roles in tumor progression. However, the effects of these three genes on different tumors are not entirely consistent. *C1QB* is associated with many cancers. According to the latest studies, the expression of *C1QB* was upregulated in CCRCC patients (Zhang et al., 2016), glioma patients (Mangogna et al., 2019b), gastric cancer patients (Jiang et al., 2020), CRC patients (Deng et al., 2022), PRCC(Mangogna et al., 2019a), and breast cancer (Mangogna et al., 2019a). And it was downregulated in nasopharyngeal carcinoma patients (Wu et al., 2020), skin cutaneous melanoma patients (Liang et al., 2022), lung adenocarcinoma (Mangogna et al., 2019a) and esophageal cancer (Yu et al., 2019). Moreover, high levels of *C1QB* expressions were associated with favorable prognosis in basal-like breast cancer (Mangogna et al., 2019a), HER2-positive breast cancer (Mangogna et al., 2019a), skin cutaneous melanoma patients (Liang et al., 2022), and osteosarcoma (Chen et al., 2021). And there was a negative correlation between higher levels of *C1QB* expression and unfavorable prognosis of patients with CCRCC(Mangogna et al., 2019a), lung adenocarcinoma (Mangogna et al., 2019a), glioma patients (Mangogna et al., 2019b), and gastric cancer patients (Jiang et al., 2020).

*C1QB* is the core promoter of the C1q molecule. An intact B chain is required for production and secretion of a serum C1q molecule (McAdam et al., 1988). The post transcriptional regulation of *C1QB* gene may affect the synthesis rate, stability and functional regulation of C1q protein, thus affecting the response process of the immune system (Yang et al., 2022). C1q is mainly produced by macrophages and dendritic cells (Verneret et al., 2014) and diffusely present in the stroma and vascular endothelium of several human malignant tumor (Bulla et al., 2016). C1q can get involved in a range of biological processes by binding to specific receptors, immune complexes, or other activators, such as angiogenesis (Bossi et al., 2014), immune modulation (Clarke and Tenner, 2014), and cell migration, adhesion, survival, and differentiation (Nayak et al., 2010). In malignant pleural mesothelioma, C1q can bind to hyaluronic acid (HA) exerts pro-tumorigenic effects (Agostinis et al., 2017b) and impacts on HA synthesis (Vidergar et al., 2021) and metabolism (Balduit et al., 2023). On the contrary, Hong et al. (2009) reported that C1q may induce apoptosis of prostate cancer cells by activating WOX1 and destabilizing cell adhesion, thus acting as an anti-tumor humoral factor. In ovarian cancer, Kaur et al. have reported that exogenous treatment with C1q and the recombinant globular head modules (gC1q) can induce apoptosis in SKOV3 cell line via TNF-α induced apoptosis pathway involving upregulation of Bax and Fas (Kaur et al., 2016). These rather two set of contradicting studies appear to suggest that the function of C1q in various tumor is complex and is strongly dependent on the TME. Recently, increasing studies based on scRNA-seq analysis have identified a distinct subset of tumor-associated macrophages (TAMs) that expresses C1q in various types of cancer. And the presence of C1q + TAM often correlates with poor prognosis. Proposed mechanisms by which C1q+ TAM drive cancer progression may correlate with exhausted T cells(Revel et al., 2022). In CRC, C1q + TAM can interact with T cell subsets via CXCL10-CXCR3 axis. This binding will activate and recruit T cells, favoring T cell exhaustion (Zhang et al., 2020). TAMs mainly originate from peripheral blood monocytes and tissue resident macrophages (Christofides et al., 2022). Thus, we infer that genetic variation in the *C1QB* may affect tissue infiltration and C1q secretion of peripheral blood monocytes, as well as the function of C1q interacting with different receptors that can influence CRC tumor progression.

With complement-targeted therapy becoming a hot topic in antitumor drug research following the detection of substantial amounts of this C component in the tumor microenvironment (Lu et al., 2021). C1q is capable of engaging a broad range of ligands via its globular domain (gC1q) which is composed of C-terminal halves of the A (ghA), B (ghB) and C (ghC) chains. Recent studies using recombinant forms of ghA, ghB and ghC have suggested that each module within the gC1q domain has a fair degree of structural and each chain can have differential ligand specificity (Kishore et al., 2004). Many C1q ligands have been found to interact in a significant way with different subunits of its globular domain (Kishore et al., 2004). In the resulting C1q model, the ghB lies on the outer part of the molecule, whereas A and C are positioned inside. It is very likely that this particular configuration has direct implications in terms of ligand recognition and C1 activation (Gaboriaud et al., 2011). For example, the ghB is considered as the principal IgG-binding module of C1q (Kishore et al., 2004). In this study, the drug target prediction and molecular docking experiments showed that the four ligands (benzo(a)pyrene, 1-naphthyl isothiocyanate, calcitriol and medroxyprogesterone acetate) were the most likely prospective drug to target the ghB. And the residues TYR:142 and LEU:220 have been implicated in the interaction. Benzo(a)pyrene, a polyaromatic hydrocarbon compound, is considered to be involved primarily in mutagenesis, carcinogenesis, and cancer metastasis promotion (Walle et al., 2006; Guo et al., 2015; Huang et al., 2020). Benzo(a)pyrene is also known to have xenoestrogenic action (Santodonato, 1997) and is currently considered to be an endocrine disruptor (Hoyer, 2001). Benzo(a)pyrene has been shown to induce apoptosis in intestinal porcine epithelial cells (Li et al., 2023a), neuronal cells (Nie et al., 2022), and endometrial cells (Yi et al., 2019). 1-Naphthyl isothiocyanate is the most widely used chemical for causing cholestasis both *in vitro* and *in vivo* according to toxicological studies (Li et al., 2016). It is secreted into bile by multidrug resistance-related proteins after hepatocyte metabolism and binding with glutathione, which has toxic effects on bile duct cells. There is currently no research on the relationship between 1-naphthyl isothiocyanate and cancer. Given the multiple side effects of benzo(a)pyrene and 1-naphthyl isothiocyanate, these agents may not be the preferred drugs for targeting C1q protein. Calcitriol is the most common biological metabolite derived from vitamin D. Deficiency of calcitriol is an epidemic that is predominantly caused by inadequate sun exposure. Calcitriol is widely used as a dietary supplement worldwide due to its beneficial effects on human health. Epidemiological studies have shown that reduced serum calcitriol levels are associated with an increased risk of CRC and a worse prognosis in CRC patients (Sluyter et al., 2021; Li et al., 2023b). Preclinical studies have revealed that the combined high-dose administration of calcitriol and certain chemotherapeutic drugs can potentiate their antitumor effects with mild side effects (Sookprasert et al., 2012; Ozgen et al., 2023). However, well-designed clinical trials are needed to optimize vitamin D administration as an anticancer therapeutic agent based on different levels of vitamin D (Henn et al., 2022). Numerous epidemiological and experimental studies have suggested the possible protective effect of medroxyprogesterone acetate on CRC risk (Chlebowski et al., 2004; Tanaka et al., 2008; Meijer et al., 2018). However, progestogens in continuous or sequential therapy used in women may be responsible for increased adverse effects both for breast cancer (Li et al., 2012), weight gain (Sims et al., 2020) and the cardiovascular system (Lizarelli et al., 2009).

This study has several advantages. First, genetic variants in *C1QB* associated with CRC were first screened, extracting data from the largest CRC risk GWAS available to the public. Second, multiple sensitivity analyses were performed to verify the dependability and coherence of the findings. Third, PPI analysis presented a promising avenue for the advancement of CRC medications via bypass. The high binding activity of the molecular docking data indicated the strong potential of *C1QB* as a drug target. Calcitriol may have the greatest advantage in targeting *C1QB* among the four drugs due to its fewer side effects.

Our study is not without limitations. There is an inconsistency between the results of MR and the analysis of the dataset GSE87211. Huiming Deng et al. reported there were significant differences in the mRNA expression of *C1QA*, *C1QB,* and *C1QC* in the TCGA colon cancer dataset. Meanwhile, the immunohistochemical staining results showed that the expression score of C1QC in CRC tissues was higher than that in non-cancer tissue. Kaplan-Meier survival analysis showed that high C1QC expression was significantly correlated with poor overall survival, which is consistent with our MR results (Deng et al., 2022). The reasons for consistent results may be as follows. Firstly, these gene variations not only affect the exposure factor *CIQB*, but may also directly or indirectly affect the outcome CRC, deviating from the actual causal relationship. Secondly, unique genetic regulatory mechanisms may exist among various tissues and the reliance on blood eQTLs for MR testing might not offer a thorough understanding of the illness and available treatment options. Thirdly, the dataset GSE87211 may contain a wider range of environmental and socio-economic factors, as well as differences in demographic variables such as race, gender, and age, leading to differences in results. Our research focused exclusively on European populations to establish genetic uniformity, this inevitably led to some limitations of the findings. Therefore, additional research and validation are needed to extend the findings to other ethnic groups with unique genetic backgrounds and confirm their wider relevance. Finally, the precision of molecular docking analysis does not necessarily translate to its effectiveness in clinical applications. Further research and verification are needed.

## 5 Conclusion

In conclusion, after performing MR analysis, we found that genetically predicted inhibition of *C1QB* may reduce the risk of CRC, which was supported by colocalization analysis. Relevant studies support the antitumor function of *C1QB*, suggesting that it is a potential drug target for CRC treatment. Additionally, drug prediction and molecular docking were used to validate the medicinal value of *C1QB*. These findings could lead to more effective CRC treatments, potentially reducing drug development costs and advancing personalized medicine approaches. However, MR analysis revealed that targeted inhibition of *C1QB* may increase the risk of ovarian cancer and melanoma. Further research and clinical trials on drugs targeting *C1QB* are warranted.

## Data Availability

The data presented in the study are deposited in the IEU Open GWAS repository (https://gwas.mrcieu.ac.uk/datasets/), accession number: finn-b-C3_COLORECTAL and eqtl-a-ENSG00000173369.
