# Supplementary material for: Causal relationship between complement C1QB and colorectal cancer: a drug target Mendelian randomization study
Source: Front Genet. 2024 Jul 23;15:1403509. doi: 10.3389/fgene.2024.1403509 (PMC11300244; doi:10.3389/fgene.2024.1403509)
Supplement: Supplementary file 1 [file Table1.DOCX]

Supplementary Material

**Supplementary Table 1 The SNPs were chosen by us as instrumental variables in forward MR analyses** **and reverse MR analyses.**

| **Exposure** | **Outcome** | **No.** | **SNP** | **Chr** | **Pos** | **E/O Allele** | **EAF** | **Beta** | **SE** | ***P*-Value** | **F** |
| --- | --- | --- | --- | --- | --- | --- | --- | --- | --- | --- | --- |
| ***The forward MR analyses*** |  |  |  |  |  |  |  |  |  |  |  |
| *C1QB* | CRC | 1 | rs141094656 | 1 | 92753336 | C/T | 0.019514 | 0.295206 | 0.042957 | 6.32E-12 | 47.2 |
| *C1QB* | CRC | 2 | rs1892548 | 13 | 41002641 | C/T | 0.638581 | -0.07446 | 0.012373 | 1.77E-09 | 36.2 |
| *C1QB* | CRC | 3 | rs2734442 | 12 | 10600368 | A/G | 0.875925 | -0.11778 | 0.018027 | 6.43E-11 | 42.7 |
| *C1QB* | CRC | 4 | rs7517504 | 1 | 22973092 | C/G | 0.077104 | 0.432316 | 0.022013 | 7.18E-86 | 385.7 |
| *C1QB* | CRC | 5 | rs7551801 | 1 | 22846452 | G/C | 0.336718 | 0.124965 | 0.01255 | 2.34E-23 | 99.1 |
| *C1QB* | CRC | 6 | rs9787086 | 1 | 22954360 | T/C | 0.290762 | 0.55709 | 0.012239 | 1E-200 | 2071.9 |
| ***The reverse MR analyses*** |  |  |  |  |  |  |  |  |  |  |  |
| CRC | *C1QB* | 1 | rs1035209 | 10 | 101345366 | T/C | 0.1609 | 0.167 | 0.0361 | 3.76201E-06 | 21.4 |
| CRC | *C1QB* | 2 | rs2736100 | 5 | 1286516 | A/C | 0.5174 | 0.1307 | 0.0266 | 9.09997E-07 | 24.1 |
| CRC | *C1QB* | 3 | rs355530 | 20 | 6404281 | T/G | 0.7118 | -0.14 | 0.0292 | 1.68298E-06 | 23.0 |

Abbreviations: SNP, single nucleotide polymorphism; Chr, chromosome; Pos, position based on GRCh37/hg19; E/O, effect/other; EAF, effect allele frequency;

SE: standard error.

**Supplementary Table 2 Mendelian randomization for** ***C1QB* on the risk of** **CRC.**

| **Outcome** | **Exposure** | **Method** | **No. of SNPs** | **Beta** | **SE** | ***P*-Value** | **OR Estimate(95% CI)** |
| --- | --- | --- | --- | --- | --- | --- | --- |
| CRC | *C1QB* | MR Egger | 6 | 0.079 | 0.073 | 0.337 | 1.083（0.939-1.249） |
|  |  | Weighted median | 6 | 0.090 | 0.049 | 0.067 | 1.094（0.994-1.205） |
|  |  | **Inverse variance weighted** | 6 | 0.099 | 0.046 | 0.033 | 1.104（1.008-1.208） |
|  |  | Simple mode | 6 | 0.124 | 0.099 | 0.265 | 1.132（0.933-1.374） |
|  |  | Weighted mode | 6 | 0.091 | 0.053 | 0.143 | 1.096（0.988-1.215） |

Abbreviations: SNP, single nucleotide polymorphism; SE, standard error; OR, odds ratio.

**Supplementary Table 3 Mendelian randomization for CRC on the risk of *C1QB*.**

| **Outcome** | **Exposure** | **Method** | **No. of SNPs** | **Beta** | **SE** | ***P*-Value** | **OR Estimate(95% CI)** |
| --- | --- | --- | --- | --- | --- | --- | --- |
| *C1QB* | CRC | MR Egger | 3 | 0.835 | 0.538 | 0.365 | 2.304（0.803-6.615） |
|  |  | Weighted median | 3 | 0.103 | 0.069 | 0.135 | 1.109（0.968-1.270） |
|  |  | Inverse variance weighted | 3 | 0.088 | 0.058 | 0.129 | 1.092（0.975-1.225） |
|  |  | Simple mode | 3 | 0.143 | 0.088 | 0.245 | 1.153（0.971-1.369） |
|  |  | Weighted mode | 3 | 0.138 | 0.087 | 0.253 | 1.148（0.968-1.361） |

Abbreviations: SNP, single nucleotide polymorphism; SE, standard error; OR, odds ratio.

**Supplementary Table 4 Pleiotropy and heterogeneity test for *C1QB* on CRC.**

|  | | | **Heterogeneity test** | | | | | | | **Pleiotropy test** | | | |
| --- | --- | --- | --- | --- | --- | --- | --- | --- | --- | --- | --- | --- | --- |
|  | | | **MR Egger** | | |  | **IVW** | | |  | **MR Egger** | | |
| **Exposure** | **Outcome** | **No. of SNPs** | **Q** | **Q_df** | **Q_pval** |  | **Q** | **Q_df** | **Q_pval** |  | **Intercept** | **SE** | ***P*** |
| *C1QB* | CRC | 6 | 1.635 | 4 | 0.803 |  | 1.754 | 5 | 0.882 |  | 0.008 | 0.023 | 0.747 |
| CRC | *C1QB* | 3 | 0.486 | 1 | 0.486 |  | 2.428 | 2 | 0.297 |  | -0.107 | 0.077 | 0.396 |

Abbreviations: SNP, single nucleotide polymorphism.


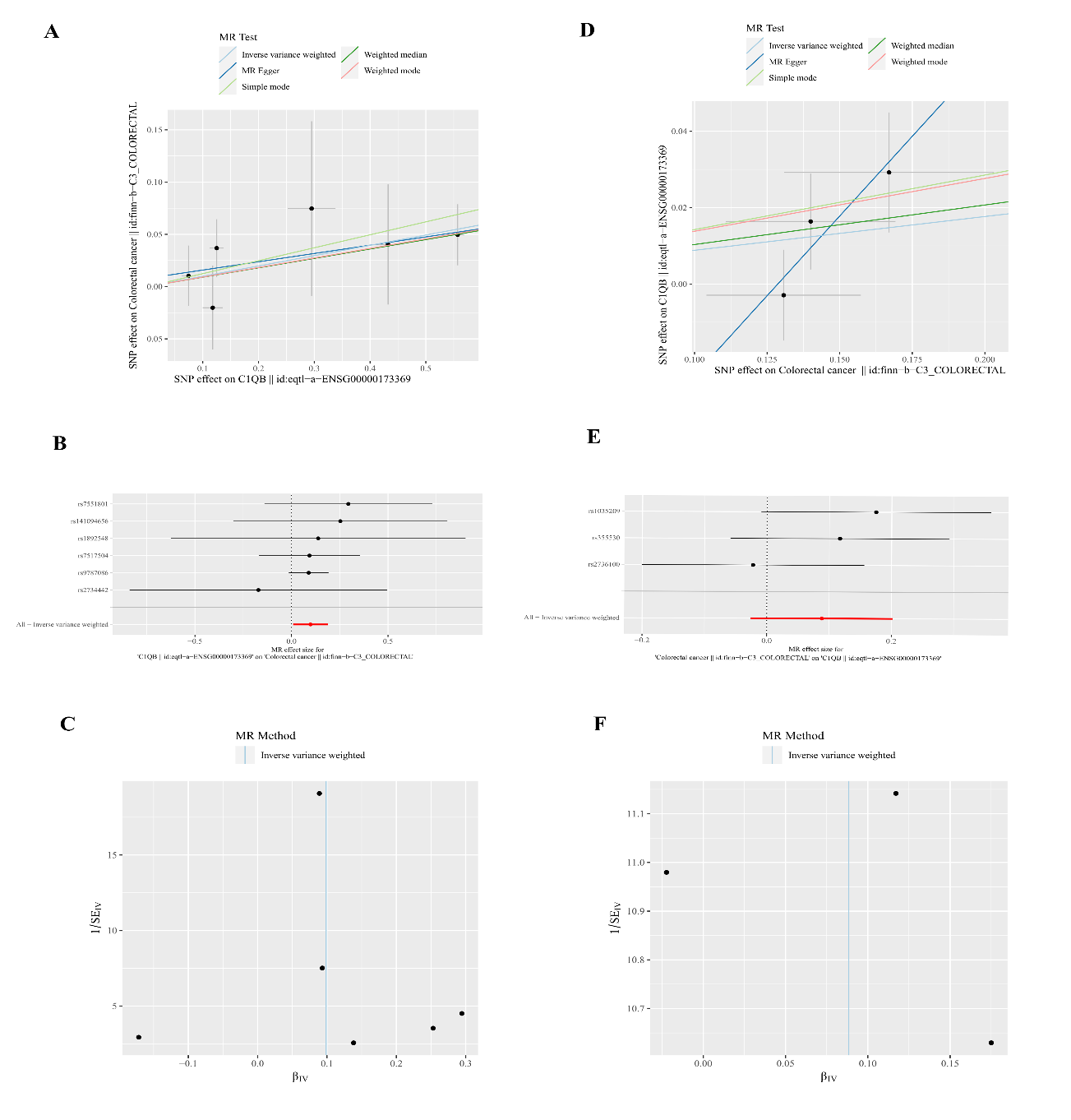


**Supplementary Figure 1.** The forward MR analyses: Casual effect of *C1QB* on CRC. (A) Scatter plot of the association between *C1QB* and CRC. The five methods applied in the current manuscript were all depicted. Lines in light blue, deep blue, light green, deep green, pink black represent inverse variance weighted, MR‐Egger, simple mode, weighted median, and weight mode methods. (B) A forest plot of the causal effects of single nucleotide polymorphisms associated with *C1QB* on CRC. The significance of red line is inverse variance weighted method. (C) A funnel plot was applied to detect whether the observed association was along with obvious heterogeneity. The reverse MR analyses: Casual effect of CRC on *C1QB*. (D) Scatter plot of the association between CRC and *C1QB*. The five methods applied in the current manuscript were all depicted. Lines in light blue, deep blue, light green, deep green, pink black represent inverse variance weighted, MR‐Egger, simple mode, weighted median, and weight mode methods. (E) A forest plot of the causal effects of single nucleotide polymorphisms associated with CRC on *C1QB*. (F) A funnel plot was applied to detect whether the observed association was along with obvious heterogeneity. Abbreviations: CRC, Colorectal cancer; MR, Mendelian randomization; SNPs, single‐nucleotide polymorphisms.


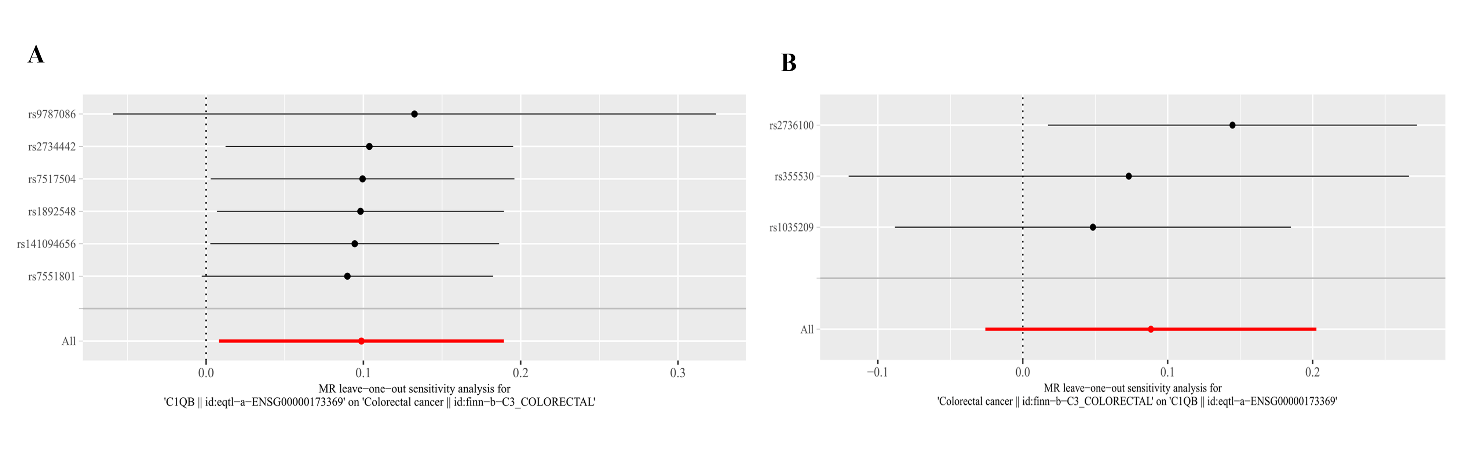


**Supplementary Figure 2** (A) The forward MR analyses: Leave‐one‐out analyses to evaluate whether any single instrumental variable was driving the causal effect. (B) The reverse MR analyses: Leave‐one‐out analyses to evaluate whether any single instrumental variable was driving the causal effect.


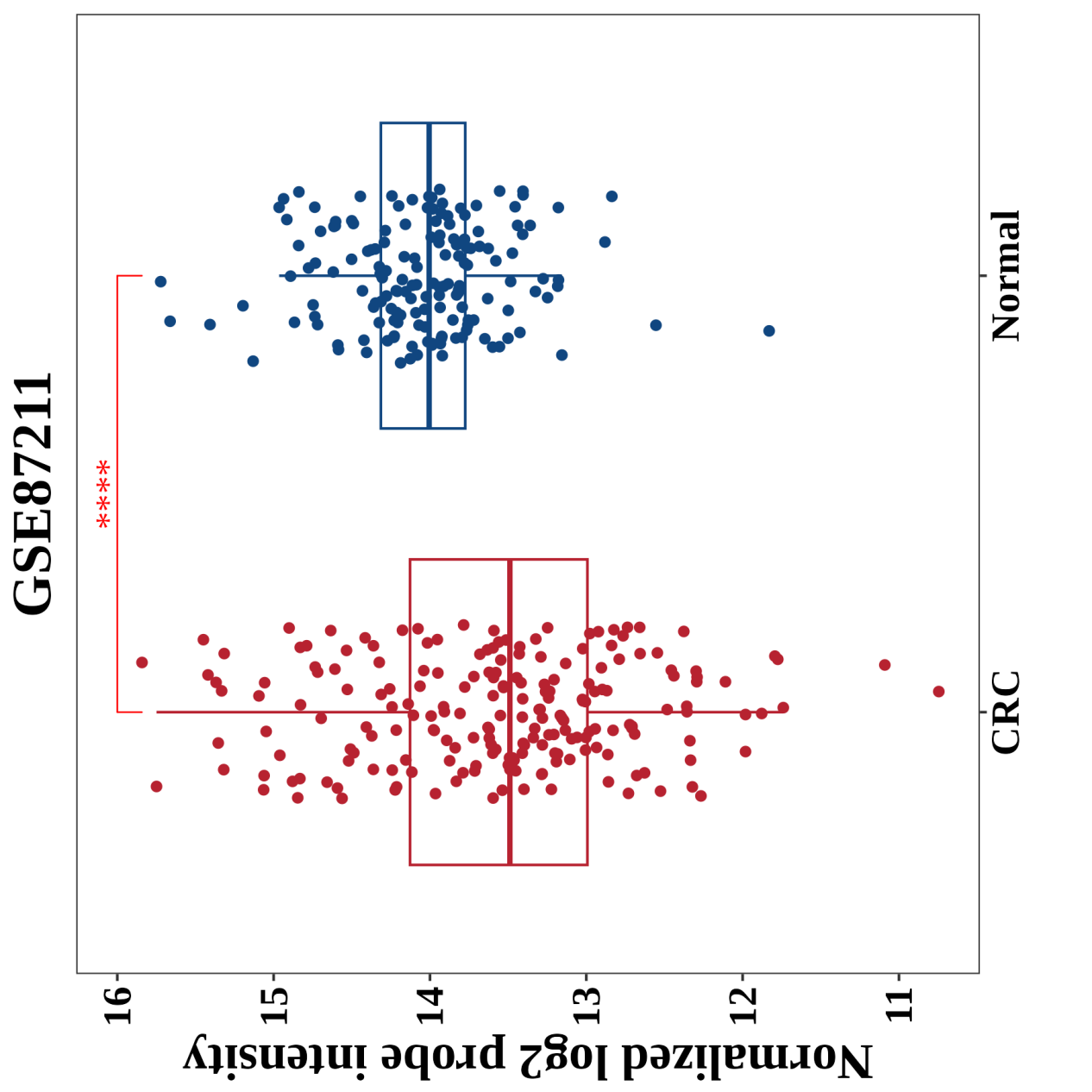


**Supplementary Figure 3**. Box plots of C1QB expressions. ^****^*P*<0.0001.
